# Higher systolic blood pressure difference in left not right upper limb is associated with all-cause mortality risk in a community-based population

**DOI:** 10.1038/s41440-025-02361-2

**Published:** 2025-09-22

**Authors:** Min Li, Fangfang Fan, Jia Jia, Jianping Li, Wei Ma, Yan Zhang

**Affiliations:** 1 Department of Cardiology, Peking University First Hospital, Beijing, China; 2 Institute of Cardiovascular Disease, Peking University First Hospital, Beijing, China; 3 State Key Laboratory of Vascular Homeostasis and Remodeling, Peking University, Beijing, China; 4 NHC Key Laboratory of Cardiovascular Molecular Biology and Regulatory Peptides, Peking University, Beijing, China; 5 Hypertension Precision Diagnosis and Treatment Research Center, Peking University First Hospital, Beijing, China

**Keywords:** Inter-arm systolic blood pressure difference, Mortality, Cohort study

## Abstract

This study investigated inter-arm systolic blood pressure difference (IASBPD) and mortality risks in 8628 Chinese community residents from an atherosclerosis cohort. IASBPD was calculated by subtracting the systolic blood pressure of the left arm from that of the right arm. Participants were categorized as |IASBPD| <10 mmHg or ≥10 mmHg. These groups were further subdivided into four categories according to specific IASBPD values. The endpoints included all-cause mortality and cardiovascular mortality. Over a median follow-up of 9.87 years, a total of 442 all-cause and 138 cardiovascular deaths were recorded. Compared to |IASBPD| <10 mmHg group, the |IASBPD| ≥10 mmHg group had a 53% increase in all-cause mortality risk (adjusted HR = 1.53, *P* = 0.002) and a 71% increase in cardiovascular mortality risk (adjusted HR = 1.71, *P* = 0.021). When comparing specific IASBPD, no significant increase in all-cause mortality risk was observed in the group with a right arm higher ≥10 mmHg (adjusted HR = 1.17, *P* = 0.507) or in the group with a left arm higher ≤10 mmHg (adjusted HR = 0.90, *P* = 0.329). Conversely, the left arm higher >10 mmHg group demonstrated a significant 59% increase in the risk of all-cause mortality (adjusted HR = 1.59, *P* = 0.013). While the association with cardiovascular mortality was not statistically significant in this subgroup, the trend paralleled that observed for all-cause mortality. In conclusion, an elevated IASBPD is significantly associated with all-cause and cardiovascular mortality risks, with higher IASBPD in the left arm showing a stronger link to all-cause mortality risk.

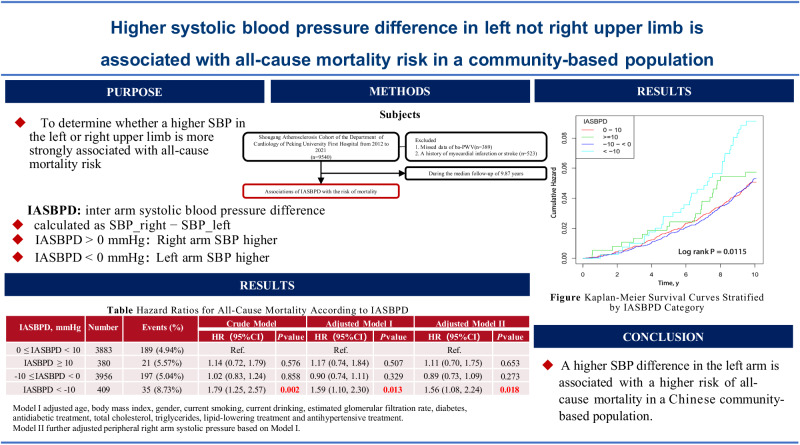

## Introduction

Hypertension constitutes a global public health burden across both developing and developed nations [1], demonstrating strong associations with diverse cardiovascular sequelae [2].Guidelines recommend the simultaneous measurement of blood pressure in both upper limbs during the initial assessment to accurately determine which arm exhibits higher blood pressure and to evaluate the inter-arm systolic blood pressure difference (IASBPD) [3]. Technological advancements in oscillometric devices have enhanced the precision and practicality of IASBPD assessment [4], transforming this metric into an emerging prognostic tool - while its diagnostic utility in peripheral artery disease screening is well-established, growing evidence supports its prognostic value for cardiovascular events and mortality risk stratification [5].

Despite over a century of scientific recognition, clinical underrecognition persists: only 13% of practitioners routinely implement bilateral measurements [6]. Contemporary research identifies IASBPD as a composite biomarker reflecting multiple cardiovascular risk determinants, including age [7], gender [8], smoking [9], alcohol consumption [9], hyperlipidemia [7, 10], and diabetes mellitus [11]. Although numerous epidemiological studies demonstrate IASBPD-mortality associations across heterogeneous populations [12–16], substantial controversy persists regarding its independent prognostic value, with conflicting reports from recent investigations [17–20]. Notably, no prospective cohort study has systematically examined the laterality-specific prognostic implications of IASBPD - a critical knowledge gap our study seeks to address.

The investigation of laterality-specific associations between IASBPD and mortality risk is grounded in distinct hemodynamic pathways. Anatomically, the left subclavian artery’s direct origin from the aortic arch renders left-arm blood pressure measurements more sensitive to proximal aortic pathology, such as atherosclerotic plaque burden or hemodynamically significant stenosis [21]. These central arterial abnormalities are established precursors of systemic vascular dysfunction. Conversely, right-arm pressure, obtained distal to the brachiocephalic trunk, may primarily reflect localized vascular changes with limited prognostic relevance.

Based on this anatomical paradigm, We hypothesized that left-arm IASBPD higher (≥10 mmHg) will demonstrate stronger associations with all-cause and cardiovascular mortality than right-arm. Therefore, the current community-based cohort study aimed to: (1) quantify mortality risk associated with directional IASBPD in a Chinese population; (2) determine whether left-arm systolic dominance confers incremental prognostic value compared to right-arm differences.

## Methods

### Study population

Participants were recruited from an atherosclerosis cohort survey conducted in the Gucheng and Pingguoyuan communities of Shijingshan district in Beijing, China, between December 2011 and April 2012. The detailed procedures of this cohort study have been previously described [22]. Initially, 9540 participants (≥40 years old) were enrolled in the baseline survey from December 2011 to April 2012, and we excluded participants without brachial-ankle pulse wave velocity (ba-PWV) measurements and those who had already reported a history of stroke or myocardial infarction at baseline. Finally, 8828 eligible subjects with a median follow-up of 9.87 years were included in this analysis. This study was approved by the ethics committee of the Peking University First Hospital, and each participant provided written informed consent. The study adhered to the principles of the Declaration of Helsinki. The procedures followed were in accordance with institutional guidelines.

### Data collection

As described in our previous report [22], all participants were interviewed by trained research coordinators using a standardized questionnaire to collect baseline data, including demographic characteristics, lifestyle, medical history, and medications. The term “current smoking” was defined as smoking one cigarette per day for at least six months. The term “Current drinking” was defined as drinking once per week for at least six months. Body mass index (BMI) was calculated as weight (kg) divided by height in meters squared (m^2^). Peripheral blood pressure (pBP) was measured using an Omron HEM-7117 electronic sphygmomanometer (Kyoto, Japan), following the standard protocol. Triplicate measurements on the right arm were performed with ≥1 min intervals between successive readings. Each patient’s peripheral SBP (pSBP) and peripheral diastolic blood pressure(pDBP) used in the analysis were calculated as the mean the three consecutive measurements.

After overnight fasting, venous blood samples were obtained from each participant by venipuncture. Serum samples were used to measure fasting blood glucose (FBG), total cholesterol (TC), triglycerides (TGs), low-density lipoprotein cholesterol (LDL-C), high-density lipoprotein cholesterol (HDL-C), and a 2-h oral glucose tolerance test (OGTT) using a Roche C8000 Automatic Analyzer. The estimated glomerular filtration rate (eGFR) was calculated using the the Chronic Kidney Disease Epidemiology Collaboration (CKD-EPI) [23].

Hypertension was defined as SBP ≥ 140 mmHg and/or DBP ≥ 90 mmHg and/or the use of anti-hypertensive medications and/or a history of hypertension. Diabetes was defined as FBG ≥ 7.0 mmol/l or/and OGTT ≥ 11.1 mmol/l or/and use of anti-diabetes medications or/and a history of diabetes. Dyslipidemia was self-reported or defined as receiving any lipid-lowering medications or concentrations of TG ≥ 1.7 mmol/L (150 mg/dL), total cholesterol (TC) ≥ 5.18 mmol/L (200 mg/dL), LDL-C ≥ 3.37 mmol/L (130 mg/dL) or HDL-C < 1.04 mmol/L (40 mg/dL). CVD was defined as any self-reported history of coronary heart disease, stroke, or transient ischemic attack.

### Measurement of inter-arm systolic blood pressure

We performed simultaneous blood pressure measurements on all four extremities while participants were in the supine position after resting for at least 5 min using an arteriosclerosis detection device (BP203RPE III, Omron Healthcare, Kyoto, Japan) by a trained technician following a standard protocol. The details of this method have been described and validated previously [24].

The inter-arm systolic blood pressure difference was calculated as the SBP of the right arm minus the SBP of the left arm. The absolute value of IASBPD, denoted by |IASBPD|, was then determined. Participants were stratified into two groups in accordance with the 2024 European Society of Hypertension Guidelines, utilizing an |IASBPD| threshold of ≥10 mmHg [25]. The IASBPD was further categorized into four distinct groups: 0–10 mmHg (right arm higher by <10 mmHg), ≥10 mmHg (right arm higher by ≥10 mmHg), −10 to <0 mmHg (left arm higher by ≤10 mmHg), and <−10 mmHg (left arm higher by >10 mmHg).

### Outcomes

Data on participant’ deaths were collected from the Chinese Center for Disease Control and Prevention (National Mortality Surveillance System) and Beijing Municipal Health Commission (Inpatient Medical Record Home Page System). The International Classification of Diseases in the 10th Revision (ICD-10) was used to classify the leading cause of death. The endpoints included all-cause death and CV death (I00-I99) [26]. We defined the follow-up time from baseline to death of the participants or the end of the follow-up (December 31, 2021).

### Statistics

For continuous variables, data are expressed as mean ± standard deviation (SD) or median (interquartile range), and categorical variables are expressed as numbers (percentages). The differences between groups were compared using the *t*-test, one-way analysis of variance (ANOVA), or Kruskal–Wallis rank test for continuous variables as appropriate, and the chi-square test or Fisher’s exact test for categorical variables as appropriate.

A generalized additive model using a spline smoothing function was employed to explore the relationship between IASBPD and mortality. Kaplan–Meier survival plots were generated to illustrate survival from baseline to the time of mortality. Univariate and multivariat Cox regression analyses were conducted to estimate the relationships between IASBPD and mortality, adjusting for potential confounders (including baseline age, body mass index, sex, current smoking, current drinking, eGFR, diabetes, antidiabetic treatment, total cholesterol, triglycerides, lipid-lowering treatment, and anti-hypertensive treatment). Additionally, the right-arm pSBP measurements were intentionally selected as the adjustment covariate because they represent the conventional clinical standard for blood pressure assessment and serve as a stable “baseline” reference.

All analyses were performed using Empower(R) (version:2.0, www.empowerstats.com, X&Y Solutions Boston, MA, USA) and R (version:3.5.1, http://www.R-project.org). A *p*-value < 0.05 (2-sided) was considered statistically significant.

## Results

### Baseline characteristics

The baseline characteristics of all participants according to IASBPD’s four groups are shown in Table 1. A total of 8628 subjects were included, with a mean age of 56.58 ± 8.97 years, and 5566 (64.51%) were female. Among them, 380 (4.40%) had an IASBPD ≥ 10 mmHg, and 409 (4.74%) had an IASBPD < −10 mmHg. Participants with higher IASBPD levels were older. They had higher BMI, pSBP, pDBP, TGs and FBG levels, as well as higher rates of hypertension, anti-hypertensive treatment, diabetes mellitus, and antidiabetic treatment.

**Table 1:** Baseline characteristics of the study participants by IASBPD four groups

	Total	Right arm higher <10 mmHg (0–10)	Right arm higher ≥10 mmHg (≥10)	Left arm higher ≤10 mmHg (−10–0)	Left higher >10 mmHg (<−10)	*P-*value
Number, *N* (%)	8628	3883 (45.00%)	380 (4.40%)	3956 (45.85%)	409 (4.74%)	
Age, y	56.58 ± 8.97	56.16 ± 8.80	57.57 ± 9.62	56.75 ± 8.99	58.02 ± 9.43	<0.001
Female, *N* (%)	5566 (64.51%)	2634 (67.83%)	298 (78.42%)	2403 (60.74%)	231 (56.48%)	<0.001
BMI, kg/m^2^	25.98 ± 3.43	25.74 ± 3.34	27.29 ± 3.74	25.91 ± 3.39	27.69 ± 3.66	<0.001
pSBP, mmHg	133.03 ± 16.66	132.05 ± 16.52	135.55 ± 16.76	133.30 ± 16.62	137.29 ± 17.34	<0.001
pDBP, mmHg	74.82 ± 9.91	74.44 ± 9.83	74.85 ± 10.03	75.08 ± 9.94	75.98 ± 10.19	0.003
Systolic pressure in right arm, mmHg	129.04 ± 16.46	129.36 ± 16.30	139.58 ± 18.32	127.66 ± 15.99	129.49 ± 16.99	<0.001
Diastolic pressure in right arm, mmHg	74.66 ± 10.35	74.72 ± 10.18	77.49 ± 10.78	74.32 ± 10.32	74.61 ± 11.37	<0.001
Systolic pressure in left arm, mmHg	129.70 ± 17.05	126.39 ± 16.18	125.13 ± 18.54	131.69 ± 16.24	146.08 ± 18.66	<0.001
Diastolic pressure in left arm, mmHg	75.15 ± 10.75	73.83 ± 10.46	71.56 ± 12.52	76.23 ± 10.32	80.68 ± 12.45	<0.001
|IASBPD|, mmHg	3.00 (2.00–6.00)	2.00 (1.00–5.00)	13.00 (11.00–16.00)	4.00 (2.00–6.00)	14.00 (12.00–18.00)	<0.001
eGFR, ml/min/1.73 m^2^	94.88 ± 13.08	95.33 ± 13.10	94.49 ± 13.39	94.62 ± 12.97	93.40 ± 13.46	0.008
Total cholesterol, mmol/L	5.34 ± 1.00	5.33 ± 1.01	5.39 ± 0.99	5.33 ± 1.00	5.35 ± 1.00	0.697
High-Density Lipoprotein Cholesterol, mmol/L	1.44 ± 0.38	1.46 ± 0.38	1.40 ± 0.34	1.43 ± 0.38	1.36 ± 0.35	<0.001
Low-Density Lipoprotein Cholesterol, mmol/L	3.26 ± 0.84	3.25 ± 0.84	3.35 ± 0.83	3.26 ± 0.83	3.29 ± 0.79	0.138
Triglycerides, mmol/L	1.30 (0.92–1.86)	1.28 (0.91–1.83)	1.33 (0.97–1.89)	1.31 (0.92–1.86)	1.41 (1.03–2.04)	<0.001
Fasting blood glucose, mmol/L	6.13 ± 1.74	6.08 ± 1.69	6.27 ± 1.67	6.14 ± 1.76	6.48 ± 2.05	<0.001
Current smoking, *N* (%)	1641 (19.02%)	693 (17.85%)	55 (14.47%)	818 (20.68%)	75 (18.34%)	0.001
Current drinking, *N* (%)	2015 (23.37%)	839 (21.62%)	60 (15.83%)	1013 (25.61%)	103 (25.18%)	<0.001
Hypertension, *N* (%)	4089 (47.39%)	1719 (44.27%)	192 (50.53%)	1945 (49.17%)	233 (56.97%)	<0.001
Antihypertensive treatment, *N* (%)	2569 (30.10%)	1062 (27.68%)	125 (33.24%)	1231 (31.43%)	151 (37.19%)	<0.001
Diabetes, *N* (%)	2065 (23.93%)	887 (22.84%)	108 (28.42%)	944 (23.86%)	126 (30.81%)	<0.001
Antidiabetic treatment, *N* (%)	839 (9.76%)	349 (9.01%)	44 (11.61%)	394 (10.00%)	52 (12.75%)	0.040
Hyperlipidemia, *N* (%)	6124 (70.98%)	2713 (69.87%)	279 (73.42%)	2830 (71.54%)	302 (73.84%)	0.130
Lipid-lowering treatment, *N* (%)	771 (9.09%)	338 (8.87%)	40 (10.61%)	363 (9.33%)	30 (7.46%)	0.418

*BMI* body mass index, *eGFR* estimated glomerular filtration rate, *IASBPD* Inter-arm systolic blood pressure difference, *pDBP* peripheral diastolic blood pressure, *pSBP* peripheral systolic blood pressure

We further analyzed the baseline characteristics of the participants using |IASBPD| (≥10 mm Hg). A total of 908 (10.52%) patients had |IASBPD| ≥10 mmHg. The median |IASBPD| was 3.00 (2.00–6.00) mmHg. The mean age of patients with |IASBPD| ≥10 mmHg was significantly greater than those with |IASBPD| <10 mmHg (57.67 ± 9.63 vs. 56.46 ± 8.88, respectively, *P* < 0.001). Individuals with |IASBPD| ≥10 mmHg were older at baseline and had higher BMI, pSBP, pDBP, TGs and FBG levels. Moreover, they presented with lower baseline eGFR and HDL-C levels. The baseline characteristics were similar to those of the four IASBPD groups (Supplementary Table S1).

### Associations of the IASBPD with the risk of mortality

During a median follow-up of 9.87 years, 442 cases of all-cause mortality (5.19%) and 138 cases of cardiovascular mortality (1.62%) occurred. Participants with |IASBPD| ≥10 mmHg exhibited significantly higher rates of all-cause mortality (7.37% vs. 4.94%, *P* = 0.002) and cardiovascular mortality (2.57% vs. 1.51%, *P* = 0.018) compared to those with |IASBPD| <10 mmHg. Higher IASBPD levels were independently associated with increased all-cause mortality risk (Supplementary Table S2).

**Fig. 1:**
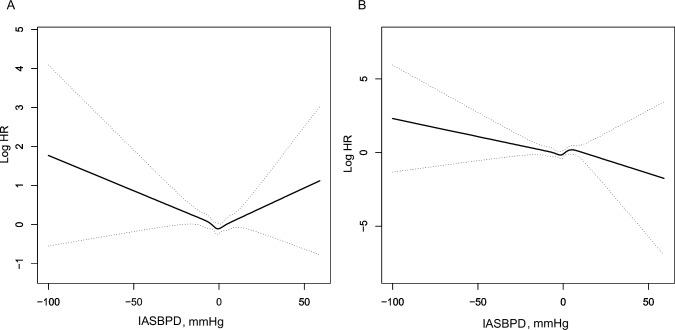
Smoothing curve showing the effects of IASBPD on risk of all-cause and cardiovascular mortality. **A** All-cause mortality; **B** Cardiovascular mortality. IASBPD Inter-arm systolic blood pressure difference, HR hazard ratio. Adjusted for age, body mass index, sex, current smoking, current drinking, estimated glomerular filtration rate, diabetes, antidiabetic treatment, total cholesterol, triglycerides, lipid-lowering treatment, and anti-hypertensive treatment

The smooth curve in Fig. 1 reflects the relationship between the IASBPD and mortality. A U-shaped curve was observed between IASBPD and all-cause mortality, It aslo showed monotonic mortality risk escalation with left-arm-dominant IASBPD magnitude versus flat trends for right-arm differences (Fig. 1A). However, the trend in the effect of IASBPD on cardiovascular mortality was nonlinear (Fig. 1B).

**Fig. 2:**
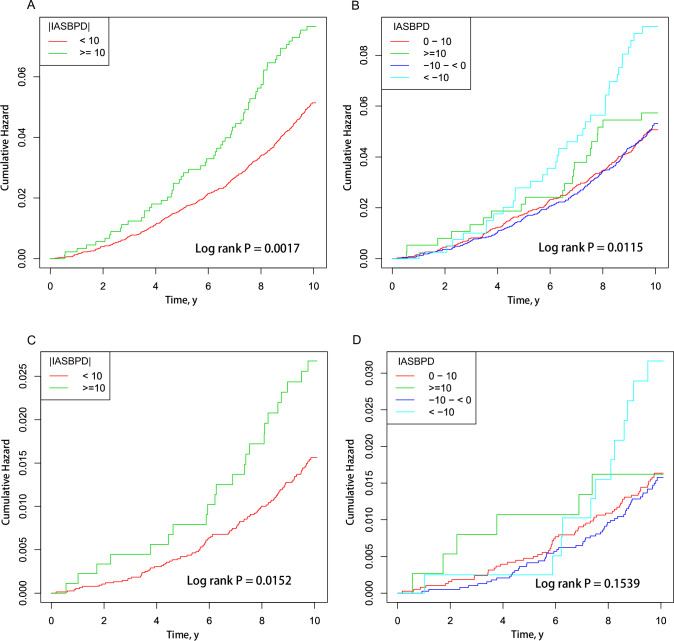
Kaplan–Meier survival curve for mortality by IASBPD. **A** Kaplan–Meier survival curve for all-cause mortality was stratified by |IASBPD| (<10 vs. ≥10 mmHg). **B** Kaplan–Meier survival curve for all-cause mortality was stratified by the four IASBPD groups. **C** Kaplan–Meier survival curve for cardiovascular mortality stratified by |IASBPD| (<10 vs ≥10 mmHg). **D** Kaplan–Meier survival curve for cardiovascular mortality stratified by IASBPD four groups. IASBPD, inter-arm systolic blood pressure difference. The *P*-value obtained using the log-rank test was used for each comparison

The Kaplan–Meier curve suggested that the greater the difference in |IASBPD|, the higher the cumulative risk of all-cause and cardiovascular mortality. The Log-rank test showed a statistically significant difference (all-cause mortality, *P* = 0.0017, cardiovascular mortality, *P* = 0.0152, Fig. 2A, C). The greater the difference in IASBPD, the higher is the cumulative risk of all-cause mortality. The Log-rank test revealed a significant difference (*P* = 0.0115, Fig. 2B). However, there was no significant difference in the cardiovascular mortality between the four groups (Fig. 2D).

Multivariate Cox Regression analysis was used to evaluate the association between IASBPD and all-cause mortality (Table 2). After adjusting for covariates, each 1 mmHg increase in |IASBPD| was associated with a 2% increase in the risk of all-cause mortality (adjust hazard ratio, HR = 1.02, 95% CI: 1.00–1.04, *P* = 0.011). Compared to the group with |IASBPD| <10 mmHg, the group with |IASBPD| ≥10 mmHg group had a 53% increase in the risk of all-cause mortality (adjusted HR = 1.53, 95% CI: 1.18 - 2.00, *P* = 0.002), and the association still existed after adjusting for pSBP. When the 0–10 mmHg (mean right arm higher <10 mmHg) group was used as the reference group, the risk of all-cause mortality was not significantly increased in the right arm higher ≥10 mmHg group (adjusted HR = 1.17, 95% CI: 0.74–1.84, *P* = 0.507) or −10–< 0 mmHg (left arm higher by ≤10 mmHg, adjusted HR = 0.90, 95%CI: 0.74–1.11, *P* = 0.329). However, the risk of all-cause mortality significantly increased by 59% in the IASBPD < -10mmHg (mean left arm higher >10 mmHg) group (adjusted HR = 1.59, 95% CI: 1.10–2.30, *P* = 0.013). The results remained significant after adjusting for pSBP (adjusted HR = 1.56, 95% CI: 1.08–2.24, *P* = 0.018).

**Table 2:** Regression models of effects of IASBPD on all-cause mortality

			Crude model		Model I^a^		Model II^b^	
	*N*	Events (%)	HR (95% CI)	*P*-Value	HR (95% CI)	*P*-Value	HR (95% CI)	*P*-Value
|IASBPD| Per 1 mmHg increment			1.02 (1.00, 1.04)	0.012	1.02 (1.00, 1.04)	0.011	1.02 (1.00, 1.04)	0.013
|IASBPD|, mmHg								
≥ 10	7720	376 (4.94%)	Reference		Reference		Reference	
< 10	908	66 (7.37%)	1.52 (1.17, 1.97)	0.002	1.53 (1.18, 2.00)	0.002	1.49 (1.14, 1.94)	0.003
IASBPD four groups, mmHg								
0–10 mmHg (right arm higher <10 mmHg)	3883	189 (4.94%)	Reference		Reference		Reference	
≥10 mmHg (right arm higher ≥10 mmHg)	380	21 (5.57%)	1.14 (0.72, 1.79)	0.576	1.17 (0.74, 1.84)	0.507	1.11 (0.70, 1.75)	0.653
−10–<0 mmHg (left arm higher ≤10 mmHg)	3956	197 (5.04%)	1.02 (0.83, 1.24)	0.858	0.90 (0.74, 1.11)	0.329	0.89 (0.73, 1.09)	0.273
<−10 mmHg (left higher >10 mmHg)	409	35 (8.73%)	1.79 (1.25, 2.57)	0.002	1.59 (1.10, 2.30)	0.013	1.56 (1.08, 2.24)	0.018

*IASBPD* Inter-arm systolic blood pressure difference, *HR* hazard ratio

^a^Model I adjusted for age, body mass index, gender, current smoking, current drinking, estimated glomerular filtration rate, diabetes, antidiabetic treatment, total cholesterol, triglycerides, lipid-lowering treatment and antihypertensive treatment

^b^Model II further adjusted peripheral systolic blood pressure based on Model I

The association between IASBPD and cardiovascular mortality is presented in Table 3. Compared to the group with |IASBPD| <10 mmHg, the group with |IASBPD| ≥10 mmHg group had a 71% increase in the risk of cardiovascular mortality (adjusted HR = 1.71, 95% CI: 1.09–2.70, *P* = 0.021), and the association still existed after adjusting for pSBP(adjusted HR = 1.59, 95% CI: 1.01–2.51, *P* = 0.047). After adjusting for covariates, there was no significant increase in the IASBPD < −10 mmHg (means left arm higher >10 mmHg) group, but the trend was consistent with all-cause mortality risk (HR = 1.58, 95% CI: 0.84–2.97, *P* = 0.155).

**Table 3:** Regression models of effects of IASBPD on cardiovascular mortality

			Crude model		Model I^a^		Model II^b^	
	*N*	Events (%)	HR (95% CI)	*P*-Value	HR (95% CI)	*P*-Value	HR (95% CI)	*P*-Value
|IASBPD| Per 1 mmHg increment			1.02 (0.99, 1.05)	0.245	1.02 (0.99, 1.05)	0.267	1.01 (0.98, 1.05)	0.375
|IASBPD|, mmHg								
≥10	7720	115 (1.51%)	Reference		Reference		Reference	
<10	908	23 (2.57%)	1.73 (1.10, 2.71)	0.017	1.71 (1.09, 2.70)	0.021	1.59 (1.01, 2.51)	0.047
IASBPD four groups, mmHg								
0–10 mmHg (right arm higher <10 mmHg)	3883	61 (1.59%)	Reference		Reference		Reference	
≥10 mmHg (right arm higher ≥10 mmHg)	380	6 (1.59%)	1.01 (0.44, 2.33)	0.987	1.00 (0.43, 2.34)	0.997	0.86 (0.37, 2.01)	0.725
−10–<0 mmHg (left arm higher ≤10 mmHg)	3956	59 (1.51%)	0.95 (0.66, 1.35)	0.757	0.77 (0.53, 1.12)	0.173	0.74 (0.51, 1.07)	0.114
<−10 mmHg (left higher >10 mmHg)	409	12 (2.99%)	1.91 (1.03, 3.55)	0.041	1.58 (0.84, 2.97)	0.155	1.48 (0.79, 2.77)	0.226

*IASBPD* Inter-arm systolic blood pressure difference, *HR* hazard ratio

^a^Model I adjusted for age, body mass index, gender, current smoking, current drinking, estimated glomerular filtration rate, diabetes, antidiabetic treatment, total cholesterol, triglycerides, lipid-lowering treatment and antihypertensive treatment

^b^Model II further adjusted peripheral systolic blood pressure based on Model I

To evaluate whether a large inter-arm systolic blood pressure difference (|IASBPD| ≥10 mmHg) is an independent prognostic factor irrespective of baseline left-arm systolic blood pressure, we performed subgroup analyses within the elevated left-arm systolic blood pressure (L-SBP ≥ 140 mmHg) and normal L-SBP (L-SBP < 140 mmHg) groups as detailed in Table S3. In the elevated L-SBP group, individuals with |IASBPD| ≥10 mmHg (*n* = 310) had a significantly higher risk of all-cause mortality compared to those with |IASBPD| <10 mmHg (*n* = 1093) after multivariable adjustment for covariates, with an adjusted HR of 1.63 (95% CI: 1.09–2.46, *P* = 0.018). Similarly, in the normal L-SBP group, individuals with |IASBPD| ≥10 mmHg (*n *= 218) exhibited a significantly elevated risk of the all-cause mortality compared to those with |IASBPD| <10 mmHg (*n* = 2744) following adjustment for the same covariates, yielding an adjusted HR of 1.93 (95% CI: 1.04–3.55, *P* = 0.036). The interaction term between L-SBP group (elevated/normal) and |IASBPD| (≥10 vs. <10 mmHg) was not statistically significant (P-interaction = 0.737), indicating that the association between IASBPD and mortality was consistent across L-SBP strata.

In analyses of the IASBPD four groups, the limited number of events (35 events in the left arm higher >10 mmHg group and 21 events in the right arm higher ≥10 mmHg group) raised concerns about model overfitting, despite the adjustment for 12 covariates. To address this, we applied least absolute shrinkage and selection operator (LASSO) regression to select the most relevant predictors from the following variables: age, body mass index, sex, current smoking, current drinking, eGFR, diabetes, antidiabetic treatment, total cholesterol, triglycerides, lipid - lowering treatment, and anti - hypertensive treatment. LASSO regression identified age, sex, eGFR, and diabetes as as the most influential predictors. These variables were subsequently used in multivariable models to reassess the association between IASBPD and all-cause mortality. The results—direction, magnitude, and statistical significance remained robust and consistent with the original fully adjusted models, as shown in Supplementary Table S4.

## Discussion

Previous research has suggested that increased IASBPD may serve as a predictor of mortality. However, the specific implications of discrepancies between SBP readings in the left and right arms have not been thoroughly investigated. Our study reveals that within populations exhibiting elevated IASBPD, individuals with a higher left arm SBP difference were associated with an increased risk of all-cause mortality. A similar trend was also observed for cardiovascular mortality.

This phenomenon can be attributed to a confluence of anatomical, hemodynamic, and pathophysiological factors. In our study cohort, the SBP was marginally higher in the left arm compared to the right, with a correspondingly elevated detection rate of IASBPD in the left arm (4.74% vs. 4.40%). This asymmetry may stem from both methodological and biological determinants. Methodologically, the larger sample size of the left-arm subgroup (*n* = 409 vs. *n* = 380) and the higher baseline SBP may have enhanced the statistical sensitivity for detecting mortality associations. Biologically, anatomical differences in arterial origins may contribute to lateralized blood pressure patterns: the right brachial artery derives from the innominate artery via the right subclavian artery, whereas the left brachial artery originates directly from the aortic arch through the left subclavian artery [27]. While this configuration typically yields slightly higher right-arm pressures physiologically, our observed left-arm SBP elevation implies potential vascular pathology. Specifically, it may indicate asymmetrical atherosclerosis, hemodynamic disturbances, or subclavian/proximal vascular dysfunction—findings that align with prior evidence positioning left-arm IASBPD as a marker of systemic vascular disease and mortality risk [28]. Mechanistically, post-hoc analyses revealed that participants with IASBPD ≥ 10 mmHg exhibited significantly higher ba-PWV compared to those with smaller differences (1747.14 ± 441.23 vs. 1633.15 ± 359.30 cm/s, *P* < 0.001). In our prior published research, we indeed observed a positive correlation between elevated IASBPD and baPWV (OR = 1.001, *P* < 0.001) [29]. These suggest that asymmetric arterial stiffening disrupts pressure propagation, particularly in aging or metabolically compromised individuals—a finding consistent with established links between aortic stiffness and peripheral pressure discordance [30, 31]. Notably, the partial mediation by ba-PWV implies coexisting mechanisms: while systemic arterial stiffening contributes to mortality risk, localized vascular pathologies (e.g., subclavian atherosclerosis) manifesting as IASBPD may independently exacerbate outcomes. This dual mechanism aligns with our observation that left-arm asymmetries correlate more strongly with atherosclerotic burden than right-arm variations [32]. However, the clinical interpretation of these findings requires caution given methodological limitations in existing literature. Most prior IASBPD studies employed sequential rather than simultaneous arm measurements, potentially confounding true pressure differences. Furthermore, the paucity of studies systematically analyzing arm-specific SBP patterns underscores the need for standardized protocols to clarify the prognostic significance of lateralized blood pressure disparities.

IASBPD have been increasingly recognized as a clinical indicator of asymmetric arterial stenosis in upper-extremity vasculature, with hemodynamic alterations typically manifesting as reduced pressure on the stenotic side. The subclavian artery represents the most prevalent site of stenosis, accounting for ~88% of such cases [4]. While our primary analysis focused on left-arm SBP. elevation as the predominant contributor to IASBPD, we acknowledge that right-arm SBP reduction—secondary to stenosis of the right subclavian or innominate artery—may also drive significant inter-arm discrepancies. Notably, only 0.13% of our cohort (*n* = 12) exhibited right-arm SBP ≤ 90 mmHg, a prevalence substantially lower than anticipated from anatomical models. This discrepancy may be attributed to the relative anatomical protection of the innominate artery compared to the left subclavian artery. Originating directly from the aortic arch, the left subclavian artery demonstrates heightened susceptibility to atherosclerotic stenosis due to its exposure to turbulent flow dynamics and mechanical stress [21]. Therefore, in our analysis, there was no significant association between the reduction in right - arm systolic blood pressure and mortality. The absence of mortality correlation with right-arm-dominant SBP elevation warrants mechanistic interpretation. First, the conventional IASBPD threshold (≥10 mmHg) may lack sensitivity for detecting right-arm-specific hemodynamic perturbations, as subtle pressure gradients caused by non-occlusive lesions or branching anomalies might evade clinical detection. Second, right-arm SBP elevation may reflect compensatory hemodynamic adjustments to anatomical variations (e.g., aberrant subclavian artery origins) rather than atherosclerotic pathology. Unlike left-sided IASBPD, which strongly correlates with aortic arch atherosclerosis, right-arm pressure discordance could be modulated by benign vascular adaptations, thereby attenuating its prognostic significance. In summary, a left-arm SBP ≥ 10 mmHg was significantly associated with increased mortality, whereas the same threshold in the right arm showed no such association. While this discrepancy may be partially attributable to statistical power, previous hemodynamic studies suggest that left-arm SBP more closely reflects central aortic pressure due to its direct anatomical connection to the aortic arch via the left subclavian artery. This lends biological plausibility to the use of left-arm SBP ≥ 10 mmHg as a more sensitive clinical risk marker. Nevertheless, this should not be interpreted to suggest that right-arm SBP differences lack pathophysiological significance.

The relationship between IASBPD and all-cause mortality remains inconsistently characterized across epidemiological studies, likely reflecting variations in cohort risk profiles and methodological approaches. While the Framingham Heart Study (*n* = 3390) found no mortality association in its low-risk, middle-aged population (HR = 1.02, 95%CI:0.76–1.38) [33]. our findings align with Shanghai cohort data (*n *= 3133) demonstrating significant risk elevation in participants [12], This divergence suggests IASBPD’s prognostic utility may be context-dependent, with heightened predictive value in populations exhibiting baseline vascular vulnerability. Our previous meta-analysis (HR = 1.28, 95% CI:0.89–1.85, *P* = 0.18) initially revealed nonsignificant pooled effects, yet substantial heterogeneity (I² = 65%, *P* = 0.01) prompted sensitivity analyses. Exclusion of outlier studies unmasked significant mortality risk for IASBPD ≥ 10 mmHg (HR = 1.43, 95% CI:1.01–2.03, *P* = 0.04) [34], underscoring the critical influence of cohort selection on observed effects. Notably, subanalyses in high-risk subgroups—including hypertensive populations [15] and patients with peripheral vascular disease [19]—consistently demonstrate IASBPD’s capacity to stratify mortality risk. These collective findings position IASBPD as a pragmatic clinical indicator, particularly valuable for risk assessment in populations with preexisting cardiovascular compromise where subtle hemodynamic disturbances carry amplified prognostic significance. In our present study, stratified analyses further demonstrated that IASBPD ≥ 10 mmHg independently predicted an increased risk of all-cause mortality in participants with both elevated (≥140 mmHg) and normal (<140 mmHg) baseline systolic blood pressure. A formal test for interaction indicated that baseline L-SBP level did not significantly modify this association (P for interaction = 0.737), suggesting that the prognostic relevance of IASBPD is consistent across blood pressure categories. Collectively, these findings support the broader applicability of a large IASBPD as a robust mortality risk marker and advocate for its inclusion in routine cardiovascular risk assessment—regardless of baseline blood pressure status.

The relationship between IASBPD and the risk of cardiovascular death remains controversial. The Vietnam experience study found no significant association between elevated IASBPD and cardiovascular mortality risk after adjusting for confounding variables [35], suggesting that IASBPD did not enhance the Framingham risk score’s predictive power regarding mortality. This finding may be limited by the relatively low prevalence of cardiovascular disease risk factors in the study cohort. Similarly, a study conducted in Shanghai reported no statistically significant difference between elevated IASBPD and cardiovascular mortality [12], which was potentially influenced by the low detection rate of elevated IASBPD (6.4%) in that study. Although some scholars argue that the effects of atherosclerosis differ between the coronary and peripheral arteries, thereby diminishing the link between IASBPD and cardiovascular mortality [36], our previous studies have shown significant correlations between IASBPD and various cardiovascular risk factors [29], suggesting that an increase in IASBPD may more accurately reflect the burden of cardiovascular risk factors. Our previous meta-analysis indicated that individuals with |IASBPD| ≥10 mmHg experienced a significantly increased risk of cardiovascular mortality compared to those with |IASBPD| <10 mmHg (HR = 1.88, 95% CI: 1.31–2.71, *P* < 0.01), with minimal heterogeneity among the studies (*P* = 0.27, I² = 23.0%) [34]. Further individual-level meta-analyses have confirmed that after adjusting for the Framingham risk score, an increase in IASBPD is associated with an increased risk of cardiovascular disease [5].

The attenuation of hazard ratios (33% reduction) upon adjustment for hypertension, diabetes, and lipid profiles in our fully adjusted models (Table 3) suggests these traditional risk factors mediate approximately one-third of IASBPD’s CVD mortality association. This aligns with mechanistic studies positioning IASBPD as an integrative marker of early vascular dysfunction preceding clinical events. Notably, the null associations with myocardial infarction (HR = 1.41, 0.92–2.18) and cerebral infarction (HR = 1.11, 0.88–1.40) in our cohort—consistent with prior reports [14, 20]—suggest IASBPD primarily reflects systemic vascular aging rather than localized atherosclerotic effects. This distinction may explain the observed mortality discordance: as a surrogate for aortic stiffness and global arterial remodeling, IASBPD’s predictive capacity emerges most clearly in studies capturing pan-vascular pathophysiology rather than isolated coronary/cerebral events.

Persisting uncertainties regarding IASBPD’s anatomical specificity (aortic vs. coronary lesions) and temporal dynamics (incident vs. progressive differences) underscore the need for targeted investigations. Future studies should incorporate advanced vascular imaging to disentangle localized plaque burden from systemic arterial degradation, while standardized IASBPD protocols (simultaneous bilateral measurements, longitudinal monitoring) could enhance clinical interpretability.

### Limitations

This study has several limitations. First, the participants were drawn from a Chinese community-based population, which raises questions about the generalizability of our findings to other populations. Second, reliance on a single measurement may be less accurate than multiple repeated assessments. Nonetheless, previous reproducibility studies have shown that as far as the BP ratio or difference is concerned, the intersession variation is small [37]. Some investigations have reported that a single reading is sufficient for screening individuals based on the IASBPD [38]. Third, although our multivariable models adjusted for 12 covariates, the relatively limited number of outcome events in subgroup analyses may have affected the precision of the effect estimates. To address this, we performed sensitivity analyses using covariate-reduced models (Supplementary Table S4), which yielded consistent results and support the robustness of our findings. Nevertheless, validation in larger and more diverse cohorts is warranted. Finally, we could not exclude patients with subclavian artery stenosis, which is anatomically related to IASBPD [39]. Future prospective studies that exclude individuals with preexisting subclavian artery disease would help clarify the impact of IASBPD on mortality risk.

## Conclusion

Our findings demonstrate that elevated IASBPD is significantly associated with the risk of all-cause and cardiovascular mortality in a Chinese community-based population, and this association remains independent of pSBP. Furthermore, individuals with a higher SBP difference in the left arm exhibited a more pronounced correlation with all-cause mortality risk.

## Supplementary information

Supplementary Tables
