# Supplementary material for: Higher systolic blood pressure difference in left not right upper limb is associated with all-cause mortality risk in a community-based population
Source: Hypertens Res. 2025 Sep 22;48(12):3187–97. doi: 10.1038/s41440-025-02361-2 (PMC12678186; doi:10.1038/s41440-025-02361-2)
Supplement: Supplementary file 1 — Supplementary Tables [file 41440_2025_2361_MOESM1_ESM.docx]

**Table S1. Baseline Characteristics of the Study Participants by |IASBPD|**

|  | **Total** | **\|IASBPD\| ≥ 10** | **\|IASBPD\| < 10** | ***P-*value** |
| --- | --- | --- | --- | --- |
| Number, N (%) | 8628 | 908 (10.52%) | 7720 (89.48%) |  |
| Age, y | 56.58 ± 8.97 | 57.67 ± 9.63 | 56.46 ± 8.88 | <0.001 |
| Female, N (%) | 5566 (64.51%) | 603 (66.41%) | 4963 (64.29%) | 0.206 |
| BMI, kg/m^2^ | 25.98 ± 3.43 | 27.38 ± 3.64 | 25.81 ± 3.37 | <0.001 |
| pSBP, mmHg | 133.03 ± 16.66 | 136.14 ± 17.50 | 132.66 ± 16.52 | <0.001 |
| pDBP, mmHg | 74.82 ± 9.91 | 75.47 ± 10.27 | 74.75 ± 9.87 | 0.040 |
| Systolic pressure in right arm, mmHg | 129.04 ± 16.46 | 133.63 ± 18.59 | 128.50 ± 16.11 | <0.001 |
| Diastolic pressure in right arm, mmHg | 74.66 ± 10.35 | 75.75 ± 11.13 | 74.53 ± 10.24 | <0.001 |
| Systolic pressure in left arm, mmHg | 129.70 ± 17.05 | 136.36 ± 21.13 | 128.91 ± 16.33 | <0.001 |
| Diastolic pressure in left arm, mmHg | 75.15 ± 10.75 | 76.54 ± 13.01 | 74.99 ± 10.45 | <0.001 |
| \|IASBPD\|, mmHg | 3.00 (2.00-6.00) | 13.00 (11.00-16.00) | 3.00 (1.00-5.00) | <0.001 |
| eGFR, ml/min/1.73m^2^ | 94.88 ± 13.08 | 93.94 ± 13.39 | 94.99 ± 13.04 | 0.023 |
| Total cholesterol, mmol/L | 5.34 ± 1.00 | 5.36 ± 0.99 | 5.33 ± 1.01 | 0.367 |
| High-Density Lipoprotein Cholesterol, mmol/L | 1.44 ± 0.38 | 1.38 ± 0.35 | 1.45 ± 0.38 | **< 0.001** |
| Low-Density Lipoprotein Cholesterol, mmol/L | 3.26 ± 0.84 | 3.31 ± 0.81 | 3.25 ± 0.84 | **0.043** |
| Triglycerides, mmol/L | 1.30 (0.92-1.86) | 1.37 (1.00-1.98) | 1.29 (0.91-1.84) | <0.001 |
| Fasting Blood Glucose, mmol/L | 6.13 ± 1.74 | 6.36 ± 1.88 | 6.11 ± 1.72 | **< 0.001** |
| Current smoking, N (%) | 1641 (19.02%) | 155 (17.07%) | 1486 (19.25%) | 0.114 |
| Current drinking, N (%) | 2015 (23.37%) | 196 (21.61%) | 1819 (23.57%) | 0.187 |
| Hypertension, N (%) | 4089 (47.39%) | 488 (53.74%) | 3601 (46.65%) | <0.001 |
| Antihypertensive treatment, N (%) | 2569 (30.10%) | 314 (34.85%) | 2255 (29.54%) | 0.001 |
| Diabetes, N (%) | 2065 (23.93%) | 261 (28.74%) | 1804 (23.37%) | <0.001 |
| Antidiabetic treatment, N (%) | 839 (9.76%) | 106 (11.71%) | 733 (9.53%) | 0.036 |
| Hyperlipidemia, N (%) | 6124 (70.98%) | 665 (73.24%) | 5459 (70.71%) | 0.113 |
| Lipid-lowering treatment, N (%) | 771 (9.09%) | 80 (8.91%) | 691 (9.11%) | 0.839 |

BMI indicates body mass index, eGFR, estimated glomerular filtration rate, IASBPD, Inter-arm systolic blood pressure difference, pDBP, peripheral diastolic blood pressure; pSBP, peripheral systolic blood pressure.

*For continuous variables, values are presented as means ± SD for normal distributed variables and median (Q1, Q3) for non-normally distributed variables.

**Table S2. Incidence of cardiovascular and all-cause mortality**

|  | **All-cause-mortality, N (%)** | **Cardiovascular mortality, N (%)** |
| --- | --- | --- |
| **\|IASBPD\|, mmHg** |  |  |
| ≥ 10 | 66 (7.37%) | 23 (2.57%) |
| < 10 | 376 (4.94%) | 115 (1.51%) |
| ***P-*value** | 0.002 | 0.018 |
| **IASBPD, mmHg** |  |  |
| 0-10 mmHg (right arm higher < 10mmHg) | 189 (4.94%) | 61 (1.59%) |
| ≥ 10mmHg (right arm higher ≥ 10mmHg) | 21 (5.57%) | 6 (1.59%) |
| -10 - < 0mmHg (left arm higher ≤ 10mmHg) | 197 (5.04%) | 59 (1.51%) |
| < -10 mmHg (left higher > 10mmHg) | 35 (8.73%) | 12 (2.99%) |
| ***P-*value** | 0.012 | 0.168 |

IASBPD, Inter-arm systolic blood pressure difference.

**Table S3. Regression Models of Effects of |IASBPD| on all-cause mortality across L-SBP Subgroups**

|  |  |  |  | **Crude Model** | | **Model I*** | |
| --- | --- | --- | --- | --- | --- | --- | --- |
| **Subgroup** | **Group** | **N** | **Events (%)** | **HR (95% CI)** | ***P*-Value** | **HR (95% CI)** | ***P*-Value** |
| **L-SBP ≥140 mmHg** | \|IASBPD\| <10 mmHg | 1093 | 92 (8.53%) |  |  | Reference |  |
|  | \|IASBPD\| ≥10 mmHg | 310 | 33 (10.96%) | 1.31 (0.88, 1.96) | 0.179 | 1.63 (1.09, 2.46) | 0.018 |
| **L-SBP <140 mmHg** | \|IASBPD\| <10 mmHg | 2744 | 95 (3.50%) | ... | ... | Reference |  |
|  | \|IASBPD\| ≥10 mmHg | 218 | 12 (5.53%) | 1.58 (0.87, 2.89) | 0.133 | 1.93 (1.04, 3.55) | 0.036 |
| **Interaction *P*-value** |  |  |  |  | 0.607 |  | 0.737 |

IASBPD, Inter-arm systolic blood pressure difference, L-SBP, left-arm systolic blood pressure HR, hazard ratio.

*Model I adjusted for age, body mass index, gender, current smoking, current drinking, estimated glomerular filtration rate, diabetes, antidiabetic treatment, total cholesterol, triglycerides, lipid-lowering treatment, antihypertensive treatment and peripheral systolic blood pressure.

**Table S4. Regression Models of Effects of IASBPD on mortality with Simplified Adjustment Variables**

|  |  |  | **Crude Model** | | **Model I*** | | **Model II^†^** | |
| --- | --- | --- | --- | --- | --- | --- | --- | --- |
|  | **N** | **Events (%)** | **HR (95% CI)** | ***P*-Value** | **HR (95% CI)** | ***P***-**Value** | **HR (95% CI)** | ***P***-**Value** |
| **All-cause mortality** |  |  |  |  |  |  |  |  |
| \|IASBPD\| Per 1mmHg increment |  |  | 1.02 (1.00, 1.04) | 0.012 | 1.02 (1.00, 1.03) | 0.034 | 1.02 (1.00, 1.03) | 0.039 |
| **\|IASBPD\|, mmHg** |  |  |  |  |  |  |  |  |
| ≥ 10 | 7720 | 376 (4.94%) | Reference |  | Reference |  | Reference |  |
| < 10 | 908 | 66 (7.37%) | 1.52 (1.17, 1.97) | 0.002 | 1.48 (1.14, 1.93) | 0.003 | 1.44 (1.11, 1.87) | 0.007 |
| **IASBPD** **four groups, mmHg** |  |  |  |  |  |  |  |  |
| 0-10 mmHg (right arm higher < 10mmHg) | 3883 | 189 (4.94%) | Reference |  | Reference |  | Reference |  |
| ≥ 10mmHg (right arm higher ≥ 10mmHg) | 380 | 21 (5.57%) | 1.14 (0.72, 1.79) | 0.576 | 1.18 (0.75, 1.85) | 0.478 | 1.15 (0.73, 1.81) | 0.535 |
| -10 - < 0mmHg (left arm higher ≤ 10mmHg) | 3956 | 197 (5.04%) | 1.02 (0.83, 1.24) | 0.858 | 0.90 (0.74, 1.10) | 0.321 | 0.92 (0.75, 1.12) | 0.401 |
| < -10 mmHg (left higher > 10mmHg) | 409 | 35 (8.73%) | 1.79 (1.25, 2.57) | 0.002 | 1.50 (1.04, 2.15) | 0.029 | 1.44 (1.01, 2.07) | 0.046 |
| **Cardiovascular mortality** |  |  |  |  |  |  |  |  |
| \|IASBPD\| Per 1mmHg increment |  |  | 1.02 (0.99, 1.05) | 0.245 | 1.01 (0.98, 1.04) | 0.361 | 1.01 (0.98, 1.05) | 0.366 |
| **\|IASBPD\|, mmHg** |  |  |  |  |  |  |  |  |
| ≥ 10 | 7720 | 115 (1.51%) | Reference |  | Reference |  | Reference |  |
| < 10 | 908 | 23 (2.57%) | 1.73 (1.10, 2.71) | 0.017 | 1.69 (1.08, 2.64) | 0.022 | 1.62 (1.04, 2.54) | 0.035 |
| **IASBPD** **four groups, mmHg** |  |  |  |  |  |  |  |  |
| 0-10 mmHg (right arm higher < 10mmHg) | 3883 | 61 (1.59%) | Reference |  | Reference |  | Reference |  |
| ≥ 10mmHg (right arm higher ≥ 10mmHg) | 380 | 6 (1.59%) | 1.01 (0.44, 2.33) | 0.987 | 1.05 (0.45, 2.45) | 0.904 | 1.03 (0.44, 2.39) | 0.946 |
| -10 - < 0mmHg (left arm higher ≤ 10mmHg) | 3956 | 59 (1.51%) | 0.95 (0.66, 1.35) | 0.757 | 0.82 (0.57, 1.17) | 0.275 | 0.84 (0.59, 1.20) | 0.343 |
| < -10 mmHg (left higher > 10mmHg) | 409 | 12 (2.99%) | 1.91 (1.03, 3.55) | 0.041 | 1.54 (0.83, 2.86) | 0.174 | 1.48 (0.79, 2.74) | 0.219 |

IASBPD, Inter-arm systolic blood pressure difference, HR, hazard ratio.

*Model I adjusted for age and gender.

^†^Model II further adjusted estimated glomerular filtration rate and diabetes based on Model I.
